# Mitigating moral distress by enhancing healthcare workers’ understanding of challenges faced by carers of children with disabilities in low-resource settings in Kenya

**DOI:** 10.1080/16549716.2025.2452159

**Published:** 2025-02-03

**Authors:** Anne Geniets, Jarim Omogi, Laura Hakimi, Alice Lakati, Niall Winters

**Affiliations:** a Department of Consultation-Liaison Psychiatry and Psychosomatic Medicine, Clinic for Survivors of Torture and War (AFK), University Hospital Zürich, Zürich, Switzerland; b Department of Medicine, University of Zürich, Zürich, Switzerland; c Amref International University, Nairobi, Kenya; d Department of Education, University of Oxford, Oxford, UK

**Keywords:** Moral distress, caregivers, community healthcare workers, children with disabilities, mental health

## Abstract

**Background:**

Little is known about the psychological wellbeing and the potential moral distress faced by female carers of children with disabilities living in low-resource settings in East Africa. In such environments, caregiving often requires resilience and resourcefulness, yet can also increase the vulnerability of caregivers and their children.

**Objective:**

The objective of this study is to identify factors affecting female caregivers’ psychological well-being, and to suggest ways healthcare workers can support these caregivers’ psychological well-being to alleviate moral distress.

**Methods:**

Employing an intersectional convergent parallel mixed-methods approach, the research explores the factors affecting the psychological wellbeing of caregivers in one urban and one rural low-resource setting in Kenya.

**Results:**

The study identifies strengthening and inhibiting factors, across three dimensions, that moderate caregivers’ experiences of moral distress, and puts forward suggestions for healthcare workers on how to support caregivers’ psychological wellbeing.

**Conclusions:**

Female carers of children with disabilities in low-resource settings in Kenya face numerous psychological, social and systemic challenges which jeopardize their caregiving, leading to moral distress. Paediatricians and nurses can contribute to enhance the caregivers’ coping-strategies and psychological well-being through simple changes, like explaining a child’s condition in non-technical language. Community health workers can help strengthen the caregivers’ already existing resources by accompanying them in the day-to-day care of their children and by helping them establish self-support groups. Consequently, improved training of healthcare- and community health workers in the field of childhood disability is needed to strengthen health systems, and to support these caregivers and their children.

## Paper Context

**Main findings:** The study identifies three dimensions of strengthening and inhibiting factors that exacerbate or mitigate the experience of moral distress of caregivers of children with disabilities living in low-income areas in Kenya and outlines opportunities for community-based healthcare interventions to support the psychological well-being of these caregivers.

**Added knowledge:** The paper adds to the existing body of knowledge by examining challenges and resources of caregivers of children with disabilities in low-income settings from an *intersectional* perspective, identifying a complex interplay of structural inequities and lack of systemic support, which exacerbate their multifactorial vulnerabilities, thereby leading to the experience of moral distress.

**Global health impact for policy and action:** The findings help to inform future global health policies and health systems strengthening initiatives in the field of child development and disability.

## Background

The aim of this study is to examine the factors which impact upon female caregivers’ psychological well-being when looking after their children with disabilities in low-resource settings in East Africa. At present, little research has examined the psychological wellbeing of these carers. This may not be surprising, given that, as Swartz and Marchetti-Mercer have argued [1], the field of disability studies is dominated by research on disability in high-income contexts.

Moral distress is defined as occurring when someone knows the morally correct action to take but is constrained from taking this action by institutional or systemic factors [2]. While it has been widely researched by healthcare professionals since Jameton’s seminal paper [3,4], the moral distress experienced by caregivers of children with disabilities in global health settings has received little to no attention. Likewise, the potential role of healthcare professionals in supporting these caregivers and alleviating their moral distress has not been sufficiently examined.

The challenges faced by caregivers of children with disabilities in low-resource settings are numerous, ranging from individual care-seeking behaviours by caregivers to more systemic and complex healthcare access challenges [5]. Perhaps unsurprisingly, caregivers from low-resource settings tend to be less likely to seek medical care for their children than caregivers from higher socio-economic environments [6]. If they do seek care, lost-to-follow up rates are high, mostly because of long waiting times and high transport costs [7,8]. Many caregivers from low-resource settings are not aware of the social and medical services available for their children with disabilities, nor of their entitlements and how to sign up for them [9,10], adding to their financial worries and contributing to their delayed care-seeking behaviour. The little research evidence that is available on the challenges for caregivers of children with disabilities in settings of chronic poverty in a global health context suggests that their physical, psychological, financial and social burdens are considerable. However, as of yet, coping strategies of caregivers and their potentially resulting moral distress in these circumstances have not received adequate attention and remain neglected issues in global health policy [11–14].

As Bury has argued [15], multidimensional studies of chronic illness and disabilities are required to understand the impact of chronic illness and disabilities on everyday life, not just in terms of stigmatisation and isolation, but also to understand the ways in which people tend to mitigate biographical disruption and to enhance adaptation. Additionally, given the gendered nature of caregiving in this setting, the intersectionality of gender and disability needs further consideration [12].

Consequently, to better understand the different factors involved, this study takes an *intersectional* perspective, investigating the factors which help these caregivers cope in their daily lives, while simultaneously examining the factors that jeopardize their caregiving, potentially leading to moral distress. Such an intersectional perspective understands a person’s lived experience as a confluence of intersecting and inter-related factors, such as being a woman, being of low socio-economic status, having low literacy, or looking after a child with a disability. These factors intersect in ways which multiply experience of vulnerability, in this case, rendering caregivers of children with disabilities potentially more vulnerable than caregivers of children without disabilities. The resulting intersecting vulnerability is often further compounded by the shortcomings of healthcare systems in resource restricted settings. Vulnerability is a widely used intersectional concept in bioethics, which describes a layered and dynamic interplay of a set of factors which render a person vulnerable, rather than just one defining feature [16,17].

So far, only a few studies have investigated the specific vulnerabilities of caregivers of children with disabilities in low-resource settings in Kenya [cf 11,12], and little to no research has focused on factors related to the healthcare system that could help mitigate these circumstances and the resulting moral distress. None of the studies has viewed the issues through an intersectional lens. This study addresses this research gap by examining the experiences of caregivers of children with disabilities in Kenya from an intersectional perspective, addressing the following research questions: *What are sources of challenges and barriers faced by caregivers looking after children with disabilities in low-resource settings?*

*What are compound effects for these caregivers and their families of living at the intersection of multiple disadvantages and multiple sources of vulnerability?*

*What are the factors that contribute to resilience for female carers of children with disabilities in low-resource settings and help mitigate various sources of vulnerabilities?*

## Methods

The context of this study emerged from a larger, completed participatory project that trained community health volunteers and their supervisors in the developmental assessment of children under five in two low-resource communities. The aims of the larger study had been to train health workers to systematically assess the development of children under five so as to contribute to early prevention and referral of children with developmental delays. As a result of the training and of the systematic assessment, a considerable number of children with developmental delays and with disabilities were identified.

### Contextualising disability services

While the Kenyan government policy on disability services is well established, the ‘on-the-ground’ reality for many marginalised children is starkly different. In the low-resource settings of this study, there were no specialised government services available for children with disabilities. Private specialist schools were out of reach financially, and forms of support for mothers were mostly restricted to self-help support groups. Many of the children had never been medically ‘diagnosed’ professionally, due to a lack of resources. This prevented anything beyond the immediate treatment of symptoms, with both support for caregivers and disability services being either non-existent in rural areas or financially unattainable.

### Study design

This study used a convergent parallel mixed-methods design to identify stress levels and sources of psycho-social support in caring for children with varying degrees of impairments in two low-resource settings, in Nairobi and in a rural county in Eastern Kenya. Qualitative data from in-depth interviews were thematically analysed and triangulated with quantitative self-assessments using the caregiver strain questionnaire (CGSQ) [18], and the Parent Experience of Child Illness (PECI) scale [19]. While the quantitative measures added important information, the main emphasis of the paper lies in the in-depth interviews undertaken with caregivers.

In order to go beyond a limited number of researcher-identified factors and to allow for cultural variation, the two quantitative self-report assessments were triangulated with findings from in-depth interviews with carers of children with disabilities. A purposive sample of 32 carers was recruited for these interviews. A COREQ checklist was used to improve transparency and reporting.

### Study location

A total of 32 carers of children with disabilities were recruited from two sites in Kenya: an urban settlement in Nairobi, in which healthcare provision is limited, poorly resourced and difficult to access, and a semi-arid, rural county in Eastern Kenya which experiences long droughts. Employment opportunities in the region are low, resulting in high poverty levels. Both locations have a low doctor to population ratio and a shortage of community health workers. Healthcare services are delivered through community health workers and health facilities but there was no focus in previous healthcare training on issues of disability.

### Measures

Two self-report measures were used to assess the caregivers’ adjustment, stress levels and resilience to their children’s impairments: the Parent Experience of Child Illness (PECI) scale [19] and the Caregiver Strain Questionnaire (CGSQ) [18]. PECI is a 25-item self-report measure that assesses carers’ adjustment to their child’s serious or chronic illness. PECI was validated in a study of 149 carers of children with brain tumors [19]. A factor analysis derived four subscales: Guilt and Worry, Emotional Resources, Unresolved Sorrow and Anger, and Long-term Uncertainty. Internal consistency for the four scales ranged from .72 to .89 [19].

CGSQ is a 21-item self-report measure of adults’ perception of difficulties associated with their parenting role. The factor structure yields three factors: Objective Burden, Externalized Subjective Burden, and Internalized Subjective Burden. Internal consistency ranged from .74 to .93 [18].

### Sampling strategy, participant recruitment and consent

Caregiver participants were identified through community health workers in both communities. In both locations, the caregivers were part of self-support groups for caregivers of children with disabilities.

The study received ethical approval both through the University of Oxford Departmental Ethics Committee and through AMREF Health Africa Ethics Review Committee. Considering the sensitivity of working with a vulnerable population, it’s crucial to ask what additional ethical considerations should be addressed prior to fieldwork, which go beyond the formal ethical procedures mandated by institutions. In our case, as experienced researchers familiar with these communities, we were well-prepared to handle any unforeseen or potentially traumatic situations that might arise, both for participants and for ourselves. Prior to beginning fieldwork, we engaged in reflective discussions on principles of meaningful participation, grounded in our belief that health equity can only be achieved through social justice.

A male and a female co-author of the paper (A.G. and J.O) interviewed the carers, assisted in translation by Kikamba-, Swahili- and English-speaking community health workers trained in working with carers of children with disabilities. Participants were given refreshments. Due to variable literacy levels, information and consent leaflets were also explained before the participants consented to ensure they understood the purpose of the study and their contribution to it.

### Participants

All of the 32 participants were caregivers of one, or in some cases, two children with varying degrees of disability and of varying age, ranging from four months old to their late teens. With the exception of two caregivers, who were the children’s grandmothers but had a primary caregiving role, all of the other women were the biological mothers of the children with a disability. In the urban participant group, most caregivers stated that their child had ‘cerebral palsy’. Only a few of the children had been formally diagnosed by healthcare workers, thus the condition was used more as an umbrella term for varying conditions. The severity of the children’s disability varied but emerged as being particularly important in this special needs’ support-restrained environment, as some caregivers described their child’s total dependence on full-time care at home, affecting their ability to look after their other children, work, travel or interact with the wider community, while other children, with some support, were able to attend a special school for children with disabilities, or a public school. Only a few of the caregivers were able to afford to send their child to a special boarding school for children with disabilities.

The children of the caregivers in the rural location seemed to have a wider range of conditions, including blindness, paralysis resulting from polio, and undiagnosed sequelae from neonatal insult and jaundice. For the protection of the participants and their children, all their names were anonymised and identifying details omitted.

### Interviews

The carers in both communities were asked to participate in in-depth interviews lasting around forty minutes. The interviews were all audio-recorded, transcribed and qualitatively analysed using NVivo software. Using content analysis of interview transcripts (both qualitative and quantitative operations on the text) [20], significant statements were extracted [21] and meanings were formulated in order to produce clusters of themes. Themes were compared within and across categories to establish consistency [22] and referred back to original interviews in order to validate them [23]. Priority was given to factors endorsed by high numbers of caregivers.

There were particular challenges relating to conducting interviews with this vulnerable group of caregivers. Almost all of the interviews were conducted in the presence of a translator. The limitations of using a translator to collect qualitative interview data are well documented [cf [24,25]. In this study, translators were also community health workers, who at times chose to respond to what the caregivers were saying with personal opinions, rather than only providing a direct translation. This may have limited or influenced the caregiver responses to the questions.

### Study limitations

While this study contributes to the understanding of the complexity of challenges faced by caregivers in low-resource settings in a global health context like Kenya, its sample is small and purposive, and the accounts of the caregivers subjective. This is appropriate for the predominantly qualitative approach and the limited time frame and resources available. Consequently, since the overall sample size is too small to allow for a generalization of the findings based on the analysis of the two self-report measures, the main contribution of the study lies in the triangulation of the quantitative and the qualitative data gathered. Finally, due to the small sample size, the study is unable discriminate between different kinds of caregiving; different care tasks (practical versus personal); and different care intensities (regular/frequent versus irregular/infrequent). For example, some of the children of caregivers needed intensive 24-hour care; whilst others were able to attend school or had short periods of illness followed by periods of relative health.

## Results

The majority of the caregivers reported high levels of stress and strain resulting from caring for their child with disability. A number of key factors were perceived by the carers to contribute to these reported stress levels. These factors, which jeopardized their caregiving, can be grouped into psychological, social and systemic dimensions. The data analysis served to identify specific inhibiting and encouraging factors within those and weighting of the evidence (Table 1).

**Table 1.** Heatmap indicating the relative strength/volume of data to support each theme/link identified below: green = strong evidence; orange = moderate evidence; red = weak evidence from this study.

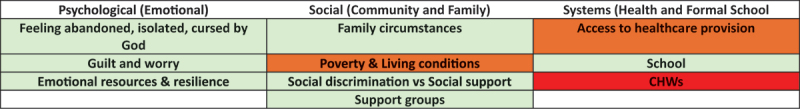	

### Psychological dimensions

Participant questionnaire responses and interview data revealed a number of related psychological factors that contributed to the mothers’ stress levels, but also feelings of emotional resilience and mastery that served as strengthening factors for the caregivers’ psychological well-being.

### Loneliness, overburden and isolation

Loneliness, which can be defined as an unwelcome feeling of lack or loss of companionship, support, and intimacy [26], emerged as an important psychological risk factor for the caregiver participants. The majority (*n* = 25; CGSQ) of questionnaire respondents reported feeling of isolation. Such feelings of isolation were often coupled with a sense that they were overburdened and thus restricted by their caregiving responsibilities.

When I am with her [caregiver’s daughter] in the house, there is nothing I can do just looking after her and if I leave her with neighbors, she will not be comfortable because she cannot feed herself, she cannot relieve herself, and she cannot talk and she only shows you with signs that she wants something. (Nairobi area, Amanda)

(…) I always pray that God only give me Medicine and School … but I do not share with anyone because when you tell someone then they go and spread it. I only talk to two women because they are also going through what I am going through by having disabled children. (Nairobi area, Jennifer)

### Guilt and worry

Several participants expressed feelings of guilt about their children’s conditions and quality of life – a finding which was supported by the questionnaire, in which 12 of the participants indicated ‘feeling guilty about the child’s disability’.

I felt so bad that I asked myself why the condition could not affect me instead of the child. I used to cry and ask ‘why my child’ as she had done no wrong to anyone … [The child] used to cry at times the whole night and she used not to eat until I wanted to end her life or [wish] if she could just die. This is because I used to feel that the child was suffering without a reason. (Nairobi area, Martha)

A minority of carers expressed a sense that the child’s disability was in some way a curse from God.

Some people see this condition as a punishment from God or you must be associated with devils or when someone help you it is as if they will get that condition. (Nairobi area, Martha)

The majority (*n* = 27; CGSQ) of participants noted they were very worried about their child’s future. ‘I get disturbed how this child’s life will be in future.’ *(Nairobi area, Godfrina)*

### Emotional resilience and resources

Despite these psychological risk factors, the carers also expressed emotional resilience and a feeling of acceptance with the situation, often aligning their own circumstances with ‘God’s plan’.

At first I withdrew myself from the community but later I accepted. (Nairobi area, Anna)

Let me say that I have left everything to God. I love my children though other people do not like them … The community should love each other and accept me because whatever happened to me is God’s plan. (Nairobi area, Lillie)

Questionnaire responses also indicate that the group of carers had a strong sense of self-reliance and a capacity to cope with their current (and future) circumstances. This can be related to the concept of mastery, defined by Pearlin as ‘the extent to which individuals view themselves as personally powerful or influential in affecting important outcomes in their lives’ [27]. However, a lesser number of carers felt at peace with their situation. While the majority of participants (*n* = 28; PECI) indicate that often or always ‘I trust myself to manage the future, whatever happens’ and that ‘I feel ready to face challenges related to my child’s wellbeing in the future’ (*n* = 27), only 10 of the participants stated ‘often or always’ for the statement ‘I am at peace with the circumstances in my life’.

### Social dimensions

Participant questionnaire responses and interview data revealed a number of social risk and protective factors. Specifically, these related to family circumstances; poverty and living conditions and the extent to which caregivers experienced discrimination or support from the surrounding community.

### Family circumstances

A minority of caregivers expressed that their family, both immediate and extended, could help to provide financial, emotional or practical support, such as the provision of childcare. In particular, a supportive spouse was highlighted as an important protective factor by a small number of participants. However, the majority of respondents report marital problems or separation and lack of support from extended family, often as a direct result of the child’s disability. Many of the caregivers also had the wider responsibility of caring for multiple siblings. All these serve as perceived risk factors for the caregivers’ psychological well-being.

‘The husband does not provide anything. He is a drunkard.’ (Rural area, Rebecca)

Yes I am married but the family does not want the child. (Nairobi area, Anna)

I used to be married but immediately [when the child] became sick, the husband left me. (Nairobi area, Ruth)

Questionnaire responses suggest that none (*n* = 0; PECI Q13) of the caregiver respondents felt they had adequate help and support available to them.

### Poverty and living conditions

Poverty and poor living conditions served as perceived inhibiting factors for caregivers and, in many cases, caregivers noted that these affected their ability to care for their children.

First the environment we are living in is so bad. For example the toilets that we use are very dirty and so because of his condition, he does not care like the bathroom is the toilet so it becomes a big challenge hence he needs a clean place that is not there. Again because at times he uses these pipes for draining urine, but at times they normally come out or the pampers. (Nairobi area, Amanda)

Some caregivers emphasised the heavy burden of their care responsibilities. In the absence of family support, or support from the wider community, the nature/intensity of daily care tasks prevented some caregivers from working regularly, thus limiting their income and their capacity to pay for the necessary healthcare and/or provide for the rest of the family. Others explained the moral distress it caused them when they had to leave the children behind in often dangerous circumstances in order to work to support the family.

At this time I cannot do much [work]and because she is also heavy, I mostly lock her up in the house so that at least I can do something. I have no one to look at the children and even if I tell the neighbors to look at the child, the neighbor laugh and even say that they are not ready to touch the child. Because where I work there is dust, I rarely go with her. (Nairobi area, Esther)

… last week I used 3000 that I had to ask form friends whom I had to call. And you know when you are the hospital and then you call a member of the family, none picks your call because they know that you want to ask for money. (Nairobi area, Martha)

Questionnaire responses highlight that almost all of the carers (*n* = 30; CGSQ Q2) *felt great financial strain as a result of their child’s disability* and were *forced to miss work or other duties* to undertake caring responsibilities.

### Social discrimination vs social support

Many caregivers describe scenarios in which disability has been misunderstood, or in which they have experienced blame, bullying or discrimination from their neighbours and wider community as a result of their child(ren)’s disability:

I: Do you feel accepted by your neighbors? R: It is hard to know that because some see disability as a communicable disease that can catch their children. (Nairobi area, Ruth)

They normally think that their children can also end up being disabled when their children join mine to play together. (Nairobi area, Lillie)

The neighbors know the problem but they normally say that he [the child] has become disabled because he has been left by the father. (Nairobi area, Esther)

Some of the neighbors are not very supportive because others think that I must have quarrelled with my neighbors and that is why I have disabled children. (Rural area, Rebecca)

The community does not like the child and whenever he passes anywhere, they say that he has stolen something but I normally told them to tell me what he has stolen so that I pay them. (Nairobi area, Anna)

However, some participants expressed the value they placed on support from their community, allowing them, for example, to work because they could leave their child with a neighbour while they did nightshifts (e.g. Nairobi area, Ruth) or providing them with reassurance and encouragement.

Actually, there was a time when I was taking the child to the clinic, people at the market were sympathising with me and there is another lady who used to give me bus fare. (Rural area, Rebecca)

I have never encountered any discrimination or insult both at the road, the neighbours and even the family members and actually, they normally encourage me that the child will be well. (Nairobi area, Judith)

### Systems dimensions

#### Access to healthcare

Timely access to healthcare and advice was a prominent factor for caregiver respondents. Many spoke positively of the care they received in hospital in the early stages of their child(ren)’s diagnosis and treatment. In a minority of cases, caregivers were able to access continued medical support and therapies at minimal cost, and this provides much reassurance:

There is somewhere here in [Nairobi area] where I normally take [the child] for physiotherapy and I am sure they normally just assist us because I am always sure that if I take [the child] there, he was definitely going to be doing the exercise and it is for free. (Nairobi area, Godfrina)

However, respondents described physical access to healthcare facilities as a particular challenge, either because those clinics and hospitals perceived as affordable were too far (more of a problem in the rural area); because it was difficult to transport the children to hospital due to their physical disabilities; or because the financial costs of the required treatment were too great to be affordable.

I: When the child is sick what do you do? R: You know now I cannot carry him on my back we have to hire a motorbike or sometimes they are expensive. We cannot carry him in the matatu. So we are just there. The only one that can help us is private but they are very expensive. From here to [the village], we cannot go with matatu (local transport system). (Rural area, Elena)

Some participants also expressed a feeling of helplessness and disempowerment when dealing with medical practitioners: a sense that ‘nothing could be done’ and that the doctor is ‘the final answer’. Often participants expressed a combination of these perceived risk factors relating to access to healthcare.

R: We were not referred, we just decided to go. We stayed there for three days and paid Ksh 21,000 and nothing much was done. I: Why do you think they did not do much at [the hospital]? R: We were not told in details what was going on. For those two days, it was just a nurse coming, looking at the kid then go away. (Rural area, Yolanda)

I: When you went to the hospital, did you ask questions? R: No. I: Why? R: Because the doctor is the final answer. (Rural area, Claudia)

### Community health workers and support groups

Only three respondents reported their visits from community health workers as a source of advice and support. But caregivers report self-help groups, or more informal contact with other caregivers for children with disabilities, as a source of emotional, financial and practical support. Examples included practical guidance – calling in expert advice/techniques for caring for child (e.g. feeding); and financial assistance (collectively paying healthcare bills; sourcing wheelchairs, etc.).

At first I did not have friends and so you find I had to have a mother who also had a disabled child that I can even ask something. That is how we came together and formed the group because that is the only person you can associate with. (Nairobi area, Martha)

There are so many people here with children with the same condition and this gives me the urge to still have my child since we share a lot with these parent. So what we did was to form a group and that is when I knew that I am not alone and I said to myself that I better stay with him because then God is with me. (Nairobi area, Edith)

At first I withdrew myself from the community but later I accepted and so nowadays I am with them. What made you go back and start mingling with them? I received counselling whenever I took the child to physiotherapy. (Nairobi area, Anna)

### School

Almost all caregivers discussed the importance of access to appropriate education for their child. While a minority of children were already attending a special school for children with disabilities (sometimes boarding), others describe their inability or reluctance to enrol their child in school due to financial burden or fear that their child would not do well. Others reported their child’s negative experiences in a mainstream school, often causing them to fall behind or drop out. Whilst school was viewed as an enabler, participants also described the emotional impact of children struggling at school.

[The child] can go to school but if she comes to this school, she is violent and she cannot beat other children. She cannot talk and hear. When she is disturbed, she normally beat children. At times, she can beat me if she feels that I have disturbed her. (Rural area, Antonia)

I: What do you think should be done to improve your situation in the community? R: If the child can be in the same school as others, then that can be comfortable to the child. I can be comfortable if the child is in school. (Rural area, Leila)

## Discussion

**Figure 1.**
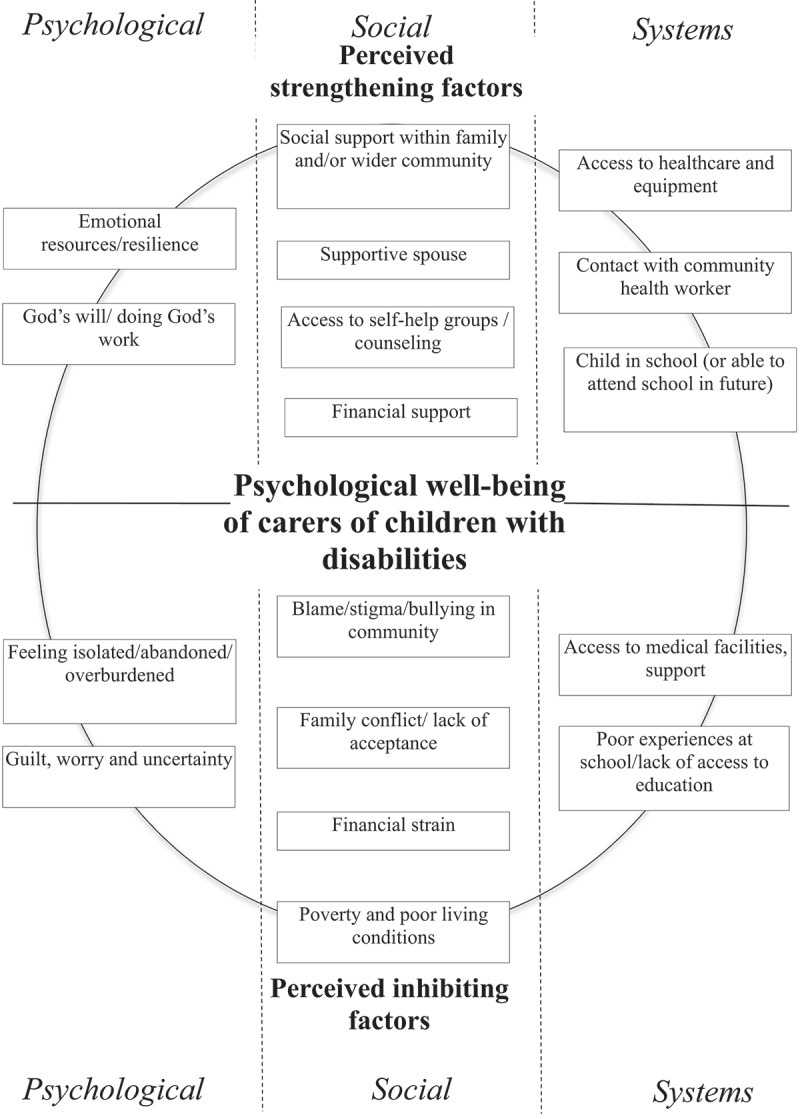
Perceived strengthening and inhibiting factors in the psychological well-being of carers of children with disabilities in low-resource settings in Kenya, which exacerbate or mitigate the experience of moral distress: a graphic summary.

As these caregivers’ experiences illustrate, the challenges of caring for children with disabilities in low-resource settings are compounded by poverty, societal stigmatization and social isolation. Lack of access to appropriate healthcare, financial strain, and lack of equipment and psycho-social support appear to additionally aggravate the stress and strain for these carers, and their children (see Figure 1). Further exacerbated by the wider societal response of blaming, shaming and stigmatization in their communities, this also seems to be the case for many of the caregivers in this study – so much so that a few of the women reported that they sometimes wished their child was dead or in difficult moments had had thoughts of harming their child. The moral distress experienced by these caregivers is clear: Having to neglect their children, leaving them at times vulnerable to abuse and danger while locked in a house, or in the care of strangers, while the caregivers have to go out to earn money, leads to further feelings of guilt and shame. However, as Scheper-Hughes in her ethnography of mothers living in extreme poverty in Brazil described, in a context where poverty-related abuse is normalized, emotions like distress are felt differently [28]. Such distress is also seen in this study but here the social construction of disability through the intersection of poverty, exclusion, and health system neglect deeply constrains caregiver agency. Consequently, for the caregivers’ children, who already display developmental delays in varying degrees, to be continuously exposed to, and potentially traumatized by, societal stigmatization, social isolation and neglect, is likely to contribute to further behavioural and developmental delays [29].

The psychological and societal impact of the children’s disabilities, and the complex interplay of structural inequalities, which appear to exacerbate the caregivers’ multifactorial vulnerabilities, seem further aggravated by a lack of systemic support. The lack of medical information about their child’s condition, its cause, aetiology, and prognosis lead to uncertainty and to self-blame, and increase the distress for the caregivers.

To interrupt this cycle, support on all levels is needed to help improve the situation for these caregivers. These include improved social and emotional support to strengthen the already existing resources, improved healthcare support, improved awareness raising about disability in communities, and improved information giving to the carers. A number of interventions can help address these issues:

First, self-support groups and peer support were mentioned by a number of women as positive means to reduce stress levels and feelings of social isolation and for sharing resources and advice. Encouraging the facilitation and usage of these groups can potentially be very beneficial, in particular for supporting the development of resilience [30] but doing so in a nuanced and informed manner that builds upon caregivers’ existing social structures and outlets.

Second, improved healthcare support could happen by including disability in the training curriculum of community health workers: Increasing the support from healthcare professionals like doctors and nurses in an already under-resourced and over-stretched healthcare system may prove difficult. Instead, community health workers could help bridging the gap by sharing information about disabilities with concerned mothers, by pointing them towards self-support groups and by supporting them in their care of their children. Simultaneously, community health workers could help raise awareness about disability in the wider community so as to address and decrease the stigmatization of carers and their children with disabilities.

## Conclusions

Intersectionality is essential to understanding the experiences of carers of children with disabilities in low- and middle-income settings, who face overlapping and compounded forms of disadvantage, and whose caregiving role intersects with other social factors such as poverty, limited access to education, healthcare systems, and disability services. Social stigma around disability often leads to isolation, discrimination, and reduced social support for these caregivers, putting them before the impossible choice of either caring for their disabled children or earning a living while neglecting them, resulting in moral distress. This study investigated factors that strengthen or inhibit the psychological well-being of caregivers of children with disabilities in two low-resource settings in Kenya in light of their intersecting disadvantages and vulnerabilities. It identified psychological, social and systemic dimensions that can exacerbate or mitigate the moral distress they experience. Understanding these intersecting challenges is key to develop inclusive policies and support systems that can address the unique needs of this population in low- and middle-income settings.

Framing theirs and their children’s lives from an intersectional perspective, the study highlights a clear need for better psychological, social and systemic support for these women and children. Paediatricians and nurses can help address this issue by making an effort to explain a child’s condition to the mother in non-technical language. Community health workers can help strengthen the caregivers’ already existing resources by accompanying them in the day-to-day care of their children and by helping them establish self-support groups. Consequently, improved communication between healthcare professionals and caregivers and enhanced training of community health workers in the field of childhood disability are needed to strengthen health systems, and to mitigate the moral distress experienced by caregivers of children with disabilities living in low resource settings.
